# Differences in Cholecystectomy Outcomes and Operating Time Between Male and Female Surgeons in Sweden

**DOI:** 10.1001/jamasurg.2023.3736

**Published:** 2023-08-30

**Authors:** My Blohm, Gabriel Sandblom, Lars Enochsson, Johanna Österberg

**Affiliations:** 1 Department of Clinical Sciences, Intervention and Technology, Karolinska Institutet, Stockholm, Sweden; 2 Department of Surgery, Mora Hospital, Mora, Sweden; 3 Center for Clinical Research, Uppsala University, Falun, Sweden; 4 Department of Clinical Science and Education, South General Hospital, Karolinska Institutet, Stockholm, Sweden; 5 Department of Surgical and Perioperative Sciences, Surgery, Umeå University, Umeå, Sweden; 6 Department of Surgery, Sunderby Hospital, Luleå, Sweden

## Abstract

**Importance:**

Female surgeons are still in the minority worldwide, and highlighting gender differences in surgery is important in understanding and reducing inequities within the surgical specialty. Studies on different surgical procedures indicate equal results, or safer outcomes, for female surgeons, but it is still unclear whether surgical outcomes of gallstone surgery differ between female and male surgeons.

**Objective:**

To examine the association of the surgeon’s gender with surgical outcomes and operating time in elective and acute care cholecystectomies.

**Design, Setting, and Participants:**

A population-based cohort study based on data from the Swedish Registry of Gallstone Surgery was performed from January 1, 2006, to December 31, 2019. The sample included all registered patients undergoing cholecystectomy in Sweden during the study period. The follow-up time was 30 days. Data analysis was performed from September 1 to September 7, 2022, and updated March 24, 2023.

**Exposure:**

The surgeon’s gender.

**Main Outcome(s) and Measure(s):**

The association between the surgeon’s gender and surgical outcomes for elective and acute care cholecystectomies was calculated with generalized estimating equations. Differences in operating time were calculated with mixed linear model analysis.

**Results:**

A total of 150 509 patients, with 97 755 (64.9%) undergoing elective cholecystectomies and 52 754 (35.1%) undergoing acute care cholecystectomies, were operated on by 2553 surgeons, including 849 (33.3%) female surgeons and 1704 (67.7%) male surgeons. Female surgeons performed fewer cholecystectomies per year and were somewhat better represented at universities and private clinics. Patients operated on by male surgeons had more surgical complications (odds ratio [OR], 1.29; 95% CI, 1.19-1.40) and total complications (OR, 1.12; 95% CI, 1.06-1.19). Male surgeons had more bile duct injuries in elective surgery (OR, 1.69; 95% CI, 1.22-2.34), but no significant difference was apparent in acute care operations. Female surgeons had significantly longer operation times. Male surgeons converted to open surgery more often than female surgeons in acute care surgery (OR, 1.22; 95% CI, 1.04-1.43), and their patients had longer hospital stays (OR, 1.21; 95% CI, 1.11-1.31). No significant difference in 30-day mortality could be demonstrated.

**Conclusions and Relevance:**

The results of this cohort study indicate that female surgeons have more favorable outcomes and operate more slowly than male surgeons in elective and acute care cholecystectomies. These findings may contribute to an increased understanding of gender differences within this surgical specialty.

## Key Points

**Question:**

Is there an association between surgeon gender and surgical outcomes in cholecystectomy?

**Findings:**

In this population-based cohort study of 150 509 patients who were operated on by 2553 surgeons in Sweden, female surgeons had significantly fewer surgical complications than male surgeons in elective and acute care cholecystectomies, including fewer bile duct injuries in elective operations. In addition, female surgeons operated more slowly; they converted to open surgery less frequently in the acute care setting; and their patients had shorter hospital stays.

**Meaning:**

These findings suggest that female and male surgeons differ in terms of surgical outcomes in elective and acute care cholecystectomies.

## Teaser

This cohort study of surgeons in Sweden examines differences between female and male surgeons in surgical outcomes and operating time in elective and acute care cholecystectomy.

## Introduction

The number of female surgeons is gradually increasing; however, they remain in the minority worldwide.^1^ In Sweden, which is considered one of the top 5 most gender-equal countries in the world,^2^ only 32% of general surgeons were female in 2020 compared to 48% of all active physicians.^3^ In comparison, the percentage of female surgeons in the UK and Japan was 27% and 22%, respectively.^4,5^

The practice of medicine is known to vary between female and male physicians,^6,7,8,9^ but less is known about whether female and male surgeons differ in surgical techniques or outcomes. A Canadian study of 25 different surgical procedures showed a slightly decreased 30-day mortality but similar surgical outcomes in patients treated by female vs male surgeons.^10^ Another study demonstrated lower mortality, fewer postoperative complications, and fewer prolonged hospital stays for patients operated on by female surgeons, but these differences disappeared when matching surgeons who worked at the same hospital.^11^ A recently published Japanese study found no difference in postoperative outcomes for female and male surgeons in major general surgery, even though female surgeons performed fewer laparoscopic procedures and operated on a higher proportion of high-risk patients.^5^

The reason behind these potential differences is still unknown. Operative technique, individual skill, and attitude most likely affect the outcome. A systematic review of gender differences in the acquisition of surgical skills concluded that male medical students had better results in simulated laparoscopy and virtual reality simulators.^12^ However, these differences did not continue for residents, as female residents seemed to respond more attentively to instructor feedback and training. Attitudes favoring competition, risk-taking behaviors, and speed could also explain gender differences.^13^ The idealization of personalities attracted by high-risk ventures, boldness, and action belongs to an abandoned era.^14^ However, a greater proportion of male medical students have been described as confident and risk-taking, whereas female medical students have longer reaction times but higher precision.^15^ The aim of this study was to examine whether female and male surgeons differ in surgical outcomes and operating time in elective and acute care cholecystectomy, one of the most frequently performed surgical procedures. These findings may contribute to an increased understanding of gender differences within this surgical specialty.

## Methods

### Study Design

The study was designed as a register-based cohort study, with data from the Swedish Registry of Gallstone Surgery and Endoscopic Retrograde Cholangiopancreatography (GallRiks). Throughout the study, the term *gender* has been used because the surgeons’ biological sex was unknown, and our research question focused on behavioral factors and attitudes. The term *sex* has been used for patients, in reference to biological sex. The study was approved by the Regional Research Ethics Committee in Uppsala, Sweden. Verbal patient consent to participate in Swedish register-based research is required for registration in GallRiks. This study followed the Strengthening the Reporting of Observational Studies in Epidemiology (STROBE) reporting guideline.^16^

### Setting and Population

All cholecystectomies registered in GallRiks between January 1, 2006, and December 31, 2019, were included in the cohort. During the study period, 162 472 patients were registered in GallRiks. After exclusions, 150 509 cholecystectomies were analyzed: 97 755 (64.9%) elective and 52 754 (35.1%) acute care operations. A flowchart of included and excluded procedures is presented in the Figure. The follow-up time was 30 days based on the registry’s organization.

**Figure.**
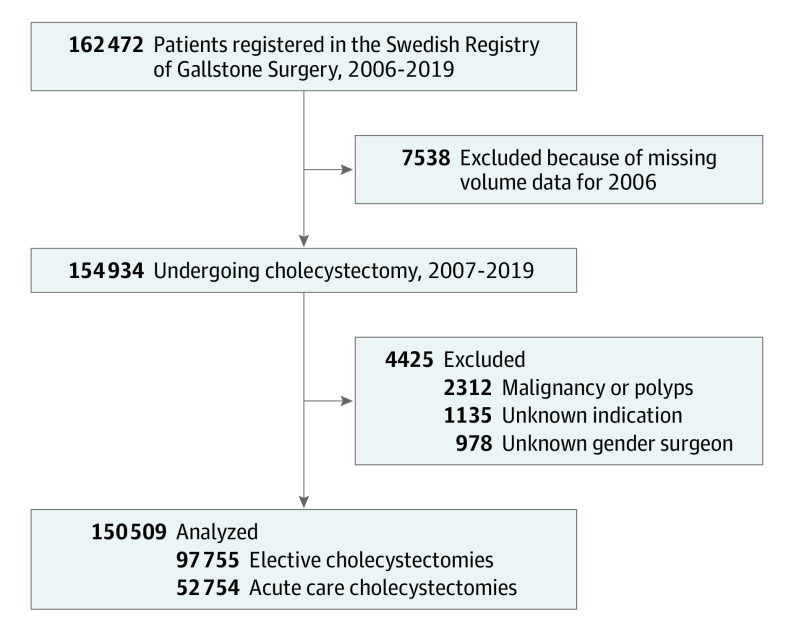
Flowchart of Included and Excluded Patients

Open and laparoscopic cholecystectomies performed on the indication of colic pain and gallstone complications (ie, cholecystitis, cholangitis, and pancreatitis) were included. Cholecystectomies as a part of surgery for malignant conditions and on the indication of gallbladder polyps were excluded. No age exclusion was made. The surgeon’s annual operative volume was calculated from the number of cholecystectomies performed the year preceding each respective procedure. Therefore, procedures from the first year (2006) were excluded from the final analyses. The cohort was described in a previous report on the importance of surgical volume in cholecystectomy.^17^

### Main Outcomes and Measures

The association between the surgeon’s gender and different outcomes was analyzed. The primary outcome was the number of surgical complications, including bleeding (requiring intervention, conversion, or blood transfusion), visceral perforation, bile duct injury (any lesion to the bile ducts other than the cystic duct), bile leakage, and abscesses. Secondary outcomes were operating time, total complications (all intraoperative and postoperative complications, such as surgical complications, thrombosis, pulmonary and cardiac complications, and wound infections), open surgery or conversion from laparoscopic to open surgery, length of stay (>3 days), and 30-day mortality. The patient’s age, sex, and American Society of Anesthesiologists (ASA) grade; previous history of acute cholecystitis; hospital type; and the surgeon’s annual operative volume were considered as potential confounders and included in the multivariable analyses. In addition, the number of days from hospital admission to surgery was included for acute care operations.

### Data Sources

GallRiks was founded in May 2005. In 2021, its national coverage was 94.5%, with a follow-up rate of 97%.^18^ The registry is financially supported by the Swedish Health Authorities and has been described in detail in previous articles.^19^ The registry includes information about patient characteristics, surgery-related parameters, and intraoperative and postoperative complications. The registry does not include specific data about the surgeons, such as age, years in practice, previous experience from other laparoscopic procedures, or information about qualities and attitudes. Approximately 14 000 cholecystectomies are registered every year in both children and adults.^18^ All surgeons in Sweden are assigned a unique identification code that remains constant even if the surgeon operates at different hospitals. Primary registration is done online by the surgeon, followed by a patient record review by a local coordinator 30 days postoperatively, to register complications. Information about 30-day mortality is obtained from the National Population Registry. Patients are informed of their registration in GallRiks when they are scheduled for surgery and can decline participation.

### Bias

The registry’s information is regularly validated by independent reviewers. Its completeness and correctness have previously been evaluated by cross-matching the registry with the Swedish National Patient Register and comparing data with medical records. This approach showed a high level of correctness with no indications of failure to report serious complications.^20^ To reduce the risk for recall bias, registrations should be performed online as soon as possible after the operation. In case of uncertainty, the register includes explanatory definitions for most variables. The local coordinators are updated regularly and trained concerning variables and postoperative adverse events.

### Statistical Analysis

Data analysis was performed from September 1 to September 7, 2022, and updated March 24, 2023. The analysis aimed at finding potential associations between the gender of the lead surgeon and different outcomes in elective and acute care cholecystectomies. The surgeon’s gender is not a variable in the registry, and gender was deduced from each surgeon’s first name. Gender data were merged into the data set based on the surgeon’s unique identification code by the national registry holder, enabling anonymization of the surgeons. Demographic characteristics of the included patients and surgeons were presented in contingency tables with difference proportions and 95% CIs. Age and surgical volumes were presented in quartile-based groups. The associations between the surgeon’s gender and risk of surgical complications, total complications, bile duct injury, conversion to open surgery, length of stay (>3 days), and 30-day mortality were calculated using logistic generalized estimating equations with exchangeable correlation structures and robust SEs. Complete cases were analyzed in the model. The results were presented as odds ratios (ORs), with 95% CIs and *P* values. Additional analysis of bleeding, thrombosis, and gallbladder perforation was performed with similar generalized estimating equations models. The association between the surgeon’s gender and operation time was calculated using a mixed linear model with the surgeon’s gender and identified confounders as fixed effects and the intercept for the surgeon, nested in hospital, as the random effect. The results were presented as the mean difference in operating time with 95% CIs and *P* values. The mean operating time, with SDs, was presented separately. The analyses included all procedures, with subgroup analyses of acute care and elective operations. A 2-sided *P* < .05 was considered significant. Statistical analysis was performed with SPSS software, version 28.0 (IBM Corp).

## Results

A total of 150 509 patients, with 97 755 (64.9%) undergoing elective cholecystectomies and 52 754 (35.1%) undergoing acute care cholecystectomies, were operated on by 2553 surgeons, including 849 (33.3%) female surgeons and 1704 (67.7%) male surgeons, at 89 registering units. Table 1 gives the patient demographic characteristics. The proportion of female surgeons increased during the study period: 489 (29.0%) female surgeons were registered in GallRiks between 2007 and 2012 compared with 651 (33.8%) between 2013 and 2019. Of the 150 509 patients, 37 847 (25.1%) were operated on by a female surgeon and 112 662 (74.9%) by a male surgeon. The mean (SD) surgical volume was 18 (15) operations per year for female surgeons and 26 (24) operations per year for male surgeons. Female surgeons were somewhat better represented at universities and private clinics. Table 2 presents the distribution of the included procedures relative to the surgeon’s gender.

**Table 1.** Demographic Characteristics of the Study Patients

Characteristic	No. (%)		Difference, % (95% CI)
	Patients in the female surgeon group (n = 37 847)	Patients in the male surgeon group (n = 112 662)	
Age, y			
<25	272 (0.7)	646 (0.6)	0.1 (0.1 to 0.2)
25-49	12 142 (32.1)	33 501 (29.7)	2.3 (1.8 to 2.9)
50-74	18 278 (48.3)	54 357 (48.3)	0.00 (−0.5 to 0.6)
≥75	7076 (18.7)	23 907 (21.2)	−2.5 (−3.0 to −2.1)
Missing	79 (0.2)	251 (0.2)	NA
Sex			
Male	12 271 (32.4)	39 030 (34.7)	−2.2 (−2.8 to −1.7)
Female	25 568 (67.6)	73 607 (65.3)	2.2 (1.7 to 2.8)
Missing	8 (0.02)	25 (0.02)	NA
ASA grade			
1	17 090 (45.2)	53 389 (47.4)	−2.3 (−2.9 to −1.7)
2-3	17 352 (45.8)	49 035 (43.5)	2.3 (1.7 to 2.9)
≥4	3314 (8.8)	9853 (8.8)	0.00 (−0.3 to 0.3)
Missing	91 (0.2)	385 (0.3)	NA

Abbreviations: ASA, American Society of Anesthesiologists; NA, not applicable.

**Table 2.** Characteristics of Included Operations and Surgeons

Characteristic	No. (%)		Difference, % (95% CI)
	Female surgeons (n = 37 847)	Male surgeons (n = 112 662)	
Surgical setting			
Acute care	12 667 (33.5)	40 087 (35.6)	−2.1 (−2.7 to −1.6)
Elective	25 180 (66.5)	72 575 (64.4)	2.1 (1.6 to 2.7)
Hospital type			
University hospital	10 197 (26.9)	25 324 (22.5)	4.5 (4.0 to 5.0)
Regional hospital	12 053 (31.8)	39 435 (35.0)	−3.2 (−3.7 to −2.6)
County hospital	11 148 (29.5)	38 146 (33.9)	−4.4 (−4.9 to −3.9)
Private clinic	4449 (11.8)	9757 (8.6)	3.1 (2.7 to 3.5)
Annual operative volume			
≤9	13 197 (34.8)	27 977 (24.8)	10.0 (9.5 to 10.6)
10-19	10 220 (27.0)	26 509 (23.5)	3.5 (3.0 to 4.0)
20-33	8384 (22.2)	26 413 (23.5)	−1.3 (−1.8 to −0.8)
>33	6046 (16.0)	31 763 (28.2)	−12.2 (−12.7 to −11.8)
Surgical access			
Laparoscopic	33 718 (89.1)	98 789 (87.7)	1.4 (1.0 to 1.8)
Laparoscopic, converted	2030 (5.4)	7229 (6.4)	−1.1 (−1.3 to −0.8)
Open surgery	1450 (3.8)	5466 (4.9)	−1.0 (−1.2 to −0.8)
Other	649 (1.7)	1178 (1.0)	0.7 (0.5 to 0.8)

### Complications

The numbers and proportions of all outcomes are presented in Table 3. Male surgeons had significantly more surgical complications (bleeding, visceral perforation, bile duct injury, postoperative bile leakage, and abscesses) in both elective (OR, 1.39; 95% CI, 1.25-1.54; *P* < .001) and acute care (OR, 1.17; 95% CI, 1.04-1.32; *P* = .01) cholecystectomies. The risk of causing a severe bile duct injury was lower for female surgeons performing elective operations, but no difference could be demonstrated in acute care operations. Patients operated on by male surgeons had significantly more total complications in both elective (OR, 1.14; 95% CI, 1.06-1.22; *P* < .001) and acute care (OR, 1.11; 95% CI, 1.02-1.20; *P* = .02) operations (Table 3).

**Table 3.** Generalized Estimating Equations for Different Outcomes Among Male and Female Surgeons

Group	All operations^a^			Elective surgery^b^				Acute care surgery^c^		
	No. (%)	OR (95% CI)	*P* value	No. (%)	OR (95% CI)	*P* value	No. (%)		OR (95% CI)	*P* value
**Surgical complications** ^d^										
Female surgeon	1258 (3.3)	1 [Reference]	NA	680 (2.7)	1 [Reference]	NA	578 (4.6)		1 [Reference]	NA
Male surgeon	4876 (4.3)	1.29 (1.19-1.40)	<.001	2650 (3.7)	1.39 (1.25-1.54)	<.001	2226 (5.6)		1.17 (1.04-1.32)	.01
**Bile duct injury** ^d^										
Female surgeon	94 (0.2)	1 [Reference]	NA	54 (0.2)	1 [Reference]	NA	40 (0.3)		1 [Reference]	NA
Male surgeon	438 (0.4)	1.56 (1.21-2.00)	<.001	254 (0.3)	1.69 (1.22-2.34)	.001	184 (0.5)		1.37 (0.93-2.00)	.11
**Total complication rate** ^e^										
Female surgeon	3401 (9.0)	1 [Reference]	NA	1924 (7.6)	1 [Reference]	NA	1477 (11.7)		1 [Reference]	NA
Male surgeon	11 527 (10.2)	1.12 (1.06-1.19)	<.001	6190 (8.5)	1.14 (1.06-1.22)	<.001	5337 (13.3)		1.11 (1.02-1.20)	.02
**Conversion to open surgery** ^d^										
Female surgeon	4129 (10.9)	1 [Reference]	NA	1777 (7.1)	1 [Reference]	NA	2352 (18.6)		1 [Reference]	NA
Male surgeon	13 873 (12.3)	1.13 (0.91-1.41)	.28	5246 (7.2)	1.03 (0.69-1.55)	.87	8627 (21.5)		1.22 (1.04-1.43)	.02
**Length of stay >3 d** ^f^										
Female surgeon	3527 (9.3)	1 [Reference]	NA	1106 (4.4)	1 [Reference]	NA	2421 (19.1)		1 [Reference]	NA
Male surgeon	12 882 (11.4)	1.21 (1.11-1.31)	<.001	3918 (5.4)	1.28 (1.14-1.45)	<.001	8964 (22.4)		1.16 (1.06-1.27)	.001
**30-d Mortality** ^d^										
Female surgeon	36 (0.1)	1 [Reference]	NA	8 (0.03)	1 [Reference]	NA	28 (0.2)		1 [Reference]	NA
Male surgeon	157 (0.1)	1.21 (0.78-1.86)	.40	30 (0.04)	1.23 (0.56-2.70)	.60	127 (0.3)		1.19 (0.74-1.92)	.48

Abbreviations: NA, not applicable; OR, odds ratio.

^a^ Adjusted for the patient’s age, sex, American Society of Anesthesiologists classification, acute care or elective surgery, previous cholecystitis, hospital type, and the surgeon’s annual operative volume.

^b^ Adjusted for the patient’s age, sex, American Society of Anesthesiologists classification, previous cholecystitis, hospital type, and the surgeon’s annual operative volume.

^c^ Adjusted for the patient’s age, sex, American Society of Anesthesiologists classification, previous cholecystitis, hospital type, the surgeon’s annual operative volume, and days in hospital before surgery.

^d^ Excluded because of missing data: all operations, 806; elective surgery, 204; and acute care surgery, 1193.

^e^ Excluded because of missing data: all operations, 4093; elective surgery, 2183; and acute surgery, 2478.

^f^ Excluded because of missing data: all operations, 3205; elective surgery, 1552; and acute care surgery, 2229.

### Operating Time

Female surgeons had significantly longer operating times in both elective and acute care cholecystectomies. The mean (SD) operating time for female surgeons was 100 (43) minutes in elective surgery and 126 (53) minutes in acute care surgery vs 89 (44) minutes in elective and 111 (55) minutes in acute care surgery for male surgeons. A mixed-model analysis of operating time found a mean difference in operating time for male surgeons compared with female surgeons of −7.96 minutes (95% CI, −9.37 to −6.54 minutes) for all operations, −6.59 minutes (95% CI, −8.07 to −5.10 minutes) for elective surgery, and −9.27 minutes (95% CI, −11.36 to −7.19 minutes) for acute care surgery (*P* < .001 for all). The mixed model gives the mean difference in operating time. The model includes patient characteristics and surgeon and hospital identification numbers, which can explain why the mean times differ from the mean difference from the mixed model.

### Conversion, Length of Stay, and Mortality

Acute care cholecystectomies performed by female surgeons were less frequently completed with the open technique or converted from laparoscopic to open surgery, but no significant difference could be demonstrated in elective surgery. Patients operated on by male surgeons had significantly longer hospital stays in both elective (OR, 1.28; 95% CI, 1.14-1.45; *P* < .001) and acute care (OR, 1.16; 95% CI, 1.06-1.27; *P* = .001) surgery. No significant difference in 30-day mortality between the genders could be demonstrated (Table 3).

### Additional Analyses

Significantly more bleeding complications were noted following procedures performed by male surgeons in elective (OR, 1.66; 95% CI, 1.24-2.23; *P* < .001) as well as acute care procedures (OR, 1.60; 95% CI, 1.15-2.21; *P* = .005). The frequency of significant bleeding complications was 97 (0.4%) in elective and 79 (0.6%) in acute care surgery for female surgeons and 463 (0.6%) in elective and 412 (1.0%) in acute care surgery for male surgeons. No difference in the number of postoperative thromboses could be demonstrated. Male surgeons registered slightly fewer iatrogenic gallbladder perforations in elective (OR, 0.90; 95% CI, 0.83-0.97; *P* = .007) and acute care surgery (OR, 0.81; 95% CI, 0.74-0.89; *P* < .001). Cholangiography is the standard routine in Sweden.^21^ A successful cholangiography was performed in 34 157 operations (90.3%) by female surgeons and 97 784 operations (86.8%) by male surgeons, with common bile duct stones identified in 4494 (11.9%) of the operations performed by female surgeons and 13 105 (11.6%) performed by male surgeons.

## Discussion

This cohort study shows that surgical outcomes in gallstone surgery differ between female and male surgeons. Female surgeons had more favorable outcomes and operated more slowly than male surgeons in both elective and acute care cholecystectomies. Unlike many other studies^5,10,11^ on gender differences in surgery, we analyzed the outcomes of a specific operation, which is performed by most surgeons, at least during their professional training. This approach made it possible to compare outcomes as well as operating times. Nevertheless, the difficulty and duration of gallstone surgery vary depending on patient characteristics, anatomical variations, timing of acute care surgery, and severity of a potential inflammation. Qualities and attitudes of the surgeon are most likely also important. Fewer complications and a longer operating time may be attributable to caution in surgical access and dissection, but these findings also mirror experience because increased operative volumes in cholecystectomies have been shown to decrease complications and the duration of surgery.^17^ The years studied, from 2007 to 2019, reflect a period when more female surgeons started their surgical careers in Sweden. Despite the longer operating times, patients of female surgeons had fewer surgical and overall complications, including bile duct injuries and bleeding.

### Comparison With Other Studies

Our study expands on the Canadian study by Wallis et al,^10^ which reported safer outcomes for female surgeons in elective surgery, because it also highlights differences in acute care procedures. In acute care surgery, the severity of the inflammation in cholecystitis and pancreatitis may affect the outcome, but in our study, female surgeons still had lower complication and conversion rates. Senior male surgeons, with limited experience in laparoscopic surgery, might contribute to the higher conversion rates in the acute care setting. Decreased 30-day mortality for patients treated by female surgeons has previously been demonstrated in elective surgery,^10^ as well as for female internists treating elderly patients.^7^ Unlike those studies, we observed no significant difference in 30-day mortality, which, in general, is low in gallstone surgery. An equal mortality rate for female and male surgeons was also observed by Tsugawa et al,^22^ who compared outcomes following 20 different emergency surgical procedures. Our results, that female surgeons have safer outcomes and operate more slowly, are consistent with a systematic review^12^ on surgical skills, which found that female medical students are slower but have higher precision. Similar outcomes but longer operating times for female surgeons have previously been observed for hysterectomies.^23^

### Interpretation and Implications

Highlighting gender differences in surgery is important in understanding inequities. In some countries where the lack of surgeons is a challenge, recruitment of more women as surgical specialists may be an efficient way to increase the workforce.^1^ Although Sweden is known for being a country with gender equity, there are still pronounced inequalities within the surgical specialty.^3^ In our study, female surgeons had lower annual volumes and slightly more elective procedures. Relatively more female surgeons worked at private clinics and universities, which may affect the distribution of acute care and elective operations. The lower annual volumes may be affected by inequities in the hospital setting, part-time work, and parental leave, especially because a greater proportion of female surgeons were starting their surgical careers during the study period.

There are various explanations for gender disparities in medicine. Studies^6,7,8,9^ have reported that female physicians adhere to guidelines more closely, use more patient-centered communication, are more willing to collaborate, and select patients for planned surgery more carefully. Personal characteristics and attitudes are difficult to study but probably affect outcomes, especially in surgery in which technical skill and decision-making are closely related to the results. This study’s important finding that female surgeons may perform safer operations and operate more slowly indicates that caution might be a favorable quality. However, it is important to highlight that competitive and risk-taking behaviors are also seen among female surgeons. We hope that our study, together with previously published studies^5,10,11^ that found that female surgeons have at least comparable outcomes as male surgeons, will encourage young female physicians to choose a surgical specialty. However, differences in surgical motivation and early results among medical students, together with the previously observed tendency that female students respond better to instructor feedback in virtual reality simulator training,^12^ stresses the importance of caution in recruitment. Continuous support and education for residents are also important,^12^ especially because lack of mentorship is a major reason why female surgeons leave the specialty.^1,24,25,26^

### Strengths and Limitations

The magnitude of the database, the register’s high national coverage of 94.5%, and the follow-up rate of 97% are strengths of the study. However, as with most register-based studies, there are obvious limitations. Data are supposed to be entered online as soon as possible after the operation, but this may vary and lead to recall bias. The register clearly states that the surgeon who performed most of the operation should be registered as the responsible surgeon. Nevertheless, it is possible that a senior colleague may be registered as the lead surgeon, even if a complication was caused by the surgeon who performed the cholecystectomy and asked for senior assistance. At least during the early years of the study period, many of the senior colleagues were male. The gender of the operating surgeon was deduced from the surgeon’s name, which might have led to errors if the name was difficult to define. However, because the Swedish surgical community is rather limited in its size, it was possible to double-check the names in case of uncertainty. Thus, it is considered unlikely that misclassification of surgeons’ gender would have substantially affected the results. All complications were included in this analysis without severity grading. The Clavien-Dindo classification^27^ was introduced as a variable in the 30-day follow-up during the later years of the study period. The patients’ other comorbidities or body mass index were not included in the multivariable analysis. Body mass index was introduced as a variable in 2010, but the information is missing in 40% of the procedures. However, body mass index is to some extent integrated into the ASA grade. We included both open and laparoscopic procedures in the cohort, which may have affected the results because open procedures are associated with more complications. The registry does not include specific data about the surgeons, such as age, years in practice, previous experience from other laparoscopic procedures, or information about qualities and attitudes, which may have helped in understanding the rationale behind the results. Identified confounders associated with both exposure and outcome have been included in the analyses. However, as for most registry-based studies, it is not possible to fully adjust for case mix, and residual confounding may still exist.

This study is based on data from GallRiks, which affects its generalizability. The Swedish health system is nationally regulated and administered regionally. To a limited extent, patients can choose their surgeon, except in some private units. The results should be interpreted with caution in countries with different cultures, gender distributions within the surgical workforce, and health economic structures. Additional observations and studies are needed to explain the gender differences more completely. Nevertheless, prioritizing thoroughness and safety, rather than speed, is an important message to bear in mind when educating younger surgeons in gallstone surgery, regardless of gender.

## Conclusions

In this population-based cohort study, female surgeons had more favorable outcomes in elective and acute care cholecystectomies and operated more slowly than male surgeons. Elective cholecystectomies were less frequently complicated by a bile duct injury when the lead surgeon was female. These findings may contribute to an increased understanding of gender differences within this surgical specialty.

## Notes

Supplement.
Data Sharing Statement
