# Supplementary material for: Differences in Cholecystectomy Outcomes and Operating Time Between Male and Female Surgeons in Sweden
Source: JAMA Surg. 2023 Aug 30;158(11):1168–75. doi: 10.1001/jamasurg.2023.3736 (PMC10469280; doi:10.1001/jamasurg.2023.3736)
Supplement: Supplement. — Data Sharing Statement [file jamasurg-e233736-s001.pdf]

Data Sharing Statement

Blohm. Differences in Cholecystectomy Outcomes and Operating Time Between Male and Female Surgeons in Sweden. *JAMA Surg*. Published August 30, 2023.
doi:10.1001/jamasurg.2023.3736

Data
Data available: Yes
Data types: Deidentified participant data
How to access data: The de-identified participant data and statistical code are available from the corresponding author (my.blohm@ki.se) with publication, upon reasonable request and after approval of the proposal by the research team.
When available: With publication

Supporting Documents
Document types: Statistical/analytic code
How to access documents: The de-identified participant data and statistical code are available from the corresponding author (my.blohm@ki.se) with publication, upon reasonable request and after approval of the proposal by the research team.
When available: With publication

Additional Information
Who can access the data: Data will be made available to researchers upon resonable request and after approval of proposal by the research team.
Types of analyses: For specified purposes, after resonable request.
Mechanisms of data availability: After approval of proposal by the research team
